# Multimodal Evaluation of Neurovascular Functionality in Early Parkinson's Disease

**DOI:** 10.3389/fneur.2020.00831

**Published:** 2020-08-26

**Authors:** Maria Marcella Laganà, Alice Pirastru, Laura Pelizzari, Federica Rossetto, Sonia Di Tella, Niels Bergsland, Raffaello Nemni, Mario Meloni, Francesca Baglio

**Affiliations:** ^1^IRCCS, Fondazione Don Carlo Gnocchi ONLUS, Milan, Italy; ^2^Department of Neurology, Buffalo Neuroimaging Analysis Center, School of Medicine and Biomedical Sciences, University at Buffalo, State University of New York, Buffalo, NY, United States; ^3^Department of Pathophysiology and Transplantation, Università degli Studi di Milano, Milan, Italy

**Keywords:** Parkinson's disease, resting state fMRI, arterial spin labeling, functional connectivity, cerebral blood flow, neurovascular coupling

## Abstract

Parkinson's disease (PD) is a multisystem neurological condition affecting different neurotransmitter pathways characterized by aberrant functional connectivity (FC) and perfusion alteration. Since the FC, measuring neuronal activity, and cerebral blood flow (CBF) are closely related through the neurovascular coupling (NVC) mechanism, we aim to assess whether FC changes found in PD mirror perfusion ones. A multimodal MRI study was implemented by acquiring resting state functional MRI (rsfMRI) and arterial spin labeling (ASL) datasets on a group of 26 early PD (66.8 ± 8 years, 22 males, median [interquartile range] Hoehn and Yahr = 1.5 [1]) and 18 age- and sex-matched healthy controls (HCs). In addition, a T1-weighted MPRAGE was also acquired in the same scan session. After a standard preprocessing, resting state networks (RSNs) and CBF maps were extracted from rsfMRI and ASL dataset, respectively. Then, by means of a dual regression algorithm performed on RSNs, a cluster of FC differences between groups was obtained and used to mask CBF maps in the subsequent voxel-wise group comparison. Furthermore, a gray matter (GM) volumetric assessment was performed within the FC cluster in order to exclude tissue atrophy as a source of functional changes. Reduced FC for a PD patient with respect to HC group was found within a sensory-motor network (SMN, p_FWE_ = 0.01) and visual networks (VNs, primary p_FWE_ = 0.022 and lateral p_FWE_ = 0.01). The latter was accompanied by a decreased CBF (primary p_FWE_ = 0.037, lateral p_FWE_ = 0.014 VNs), while no GM atrophy was detected instead. The FC alteration found in the SMN of PD might be likely due to a dopaminergic denervation of the striatal pathways causing a functional disconnection. On the other hand, the changes in connectivity depicted in VNs might be related to an altered non-dopaminergic system, since perfusion was also reduced, revealing a compromised NVC. Finally, the absence of GM volume loss might imply that functional changes may potentially anticipate neurodegeneration. In this framework, FC and CBF might be proposed as early functional biomarkers providing meaningful insights in evaluating both disease progression and therapeutic/rehabilitation treatment outcome.

## Introduction

Parkinson's disease (PD) is one of the most frequent neurodegenerative disorders affecting over four million people worldwide (1). PD is clinically characterized by both motor symptoms, such as tremor, bradykinesia, and rigidity, and non-motor ones, including cognitive impairment, neuropsychiatric symptoms, and autonomic dysfunction (2, 3). From a neuropathological point of view, PD can be considered as a multisystem brain disorder (4, 5) affecting different neurotransmitter pathways. The dopaminergic denervation of striatal pathways is considered the cardinal signature of PD, and it is often linked to motor symptoms (5). The other pathophysiological feature of PD is the progressive deposition of α-synuclein in cholinergic and monoaminergic brain neurons, concurrent with the evolution of Lewy body pathology (6).

The scientific community has shown a great interest in trying to identify non-invasive imaging biomarkers that may improve our understanding of the mechanisms underlying PD. In this framework, both resting state functional magnetic resonance imaging (rsfMRI) and arterial spin labeling (ASL) have provided considerable insights into the neural correlates of PD, detecting functional connectivity (FC) and cerebral blood flow (CBF) alterations, respectively.

FC alterations in PD have been extensively reported both in terms of increased and decreased connectivity (7, 8). Increased blood oxygen level dependent (BOLD) signal was found in primary and secondary motor cortices and the middle frontal gyrus of PD patients (9). On the other hand, decreased FC was observed in the supplementary motor area (SMA) (10) and between temporal regions and left occipital cortex and left lingual gyrus (11). Furthermore, reduced FC in posterior cortical regions has been associated with global cognitive decline (12), while the disruption of anticorrelation patterns between the occipito-parietal areas and the default mode network correlated with visuospatial deficits in PD (13).

Besides FC changes, perfusion alterations were also observed in PD (14, 15). Perfusion was found to be reduced in pre-SMA (16). Reduced CBF was also reported in occipital and parietal cortices (14, 17), precuneus and cuneus (17), and frontal cortex (16) bilaterally. Hypoperfusion was hypothesized to be related to the alteration of cholinergic, serotoninergic, and noradrenergic neurotransmitter pathways in PD (16, 18, 19). Although several studies reported no perfusion changes in PD (18, 20, 21), in Pelizzari et al. (21), the resulting CBF correlated with symptoms severity, while Al-Bachari et al. (18) revealed a prolonged arterial arrival time confirming an aberrant neurovascular status of PD.

Since BOLD signal reflects changes in the venous oxygenation level (22), rsfMRI contrast is closely dependent on CBF (23). The neuronal activity and cerebral perfusion are strictly related by means of the physiological mechanism known as neurovascular coupling (NVC), which enables the prompt adaptation of brain perfusion to the (local) metabolic demand. Evidence of injury to both neural innervations and capillaries were reported in idiopathic PD (19), suggesting that the neurovascular unit is affected at different levels in PD. For these reasons, cross-talk between observed FC and CBF alterations in PD cannot be excluded.

In order to better understand the relationship between neural activity and perfusion alterations in PD, we conducted a multimodal MRI study by concurrently assessing FC and CBF by means of rsfMRI and ASL, respectively. We aimed to investigate whether the FC changes found in PD reflect perfusion alterations. Gray matter (GM) volume was also assessed to exclude atrophy as a confounding factor of functional and perfusion changes.

## Methods

### Demographic and Clinical Evaluation

Twenty-six PD patients and 18 age and sex-matched healthy controls (HC) were enrolled in this study. The inclusion criteria for the PD patients were as follows: (1) a diagnosis of idiopathic PD according to the Movement Disorder Society Clinical Diagnostic Criteria for PD (24); (2) absence of neuropsychiatric disorders beside PD diagnosis at clinical evaluation; (3) absence of neurovascular diseases at clinical evaluation, documented also with previous MRI/CT examination; (4) Positive DaTscan; (5) mild to moderate stage of the disease with a scoring between stages 1 and 3 of the Modified Hoehn and Yahr (H&Y) Scale (25); (6) stable drug therapy with either L-Dopa or dopamine agonists; (7) freezing assessed with UPDRS part II lower than 2; and (8) time spent with dyskinesias assessed with UPDRS part IV lower than 2. HC were included after assessing the absence of any neurological and/or neuropsychiatric disorder and/or neurovascular diseases.

All participants were right-handed.

For PD patients, the clinical evaluation included the quantification of the disease stage with H&Y and the assessment of the symptom severity with UPDRS motor part III (UPDRS III) performed by an experienced neurologist. Moreover, PD patients were classified as either tremor dominant or akinetic-rigid (26). Drug administration was recorded, and levodopa equivalent daily dose (LEDD) was calculated as suggested in Tomlinson et al. (27).

The global cognitive level of all the participants was assessed with the Montreal Cognitive Assessment (MoCA) test.

The study was performed in accordance with the principles of the Helsinki Declaration and by previous approval from the IRCSS Fondazione Don Carlo Gnocchi Ethics Committee. Written informed consent was signed by each participant.

### MRI Acquisition

All the subjects underwent a magnetic resonance imaging (MRI) examination performed on a 1.5T Siemens Avanto scanner (Erlangen, Germany) equipped with a 12-channel head coil. The acquisition comprised: (1) a 3D high-resolution magnetization-prepared rapid gradient echo (MPRAGE) T1-weighted image [repetition time (TR) = 1,900 ms, echo time (TE) = 3.3 ms, inversion time (TI) = 1,100 ms, matrix size = 192 × 256 × 176, resolution = 1 mm^3^ isotropic]; (2) a multi-echo resting state fMRI (ME-rsfMRI) sequence (TR = 2,570 ms, TE = 15/34/54 ms, matrix size = 64 × 64 × 31, resolution = 3.75 × 3.75 × 4.5 mm^3^, 200 volumes); (3) a double-echo GRE field map (TR = 528 ms, TE = 4.76/9.52 ms, matrix size = 100 × 100 × 42, resolution = 3.2 × 3.2 × 3.3 mm^3^); and (4) a 3D gradient and spin echo (GRASE) multidelay pseudo-continuous arterial spin labeling (pCASL) with background suppression sequence [TR/TE = 3,500/22.58 ms, labeling duration = 1,500 ms, 5 post-labeling delays (PLD) = 700/1200/1700/2200/2700 ms, 12 pairs of tag/control volumes, matrix size = 64 × 64 × 32, resolution = 3.5 × 3.5 × 5 mm^3^, distance between the center of imaging slices and labeling plane = 90 mm].

### MRI Analysis

The image processing was performed by means of FMRIB Software Library (FSL, http://www.fmrib.ox.ac.uk/fsl) toolboxes 5.0.6 if not otherwise specified.

**Figure 1:**
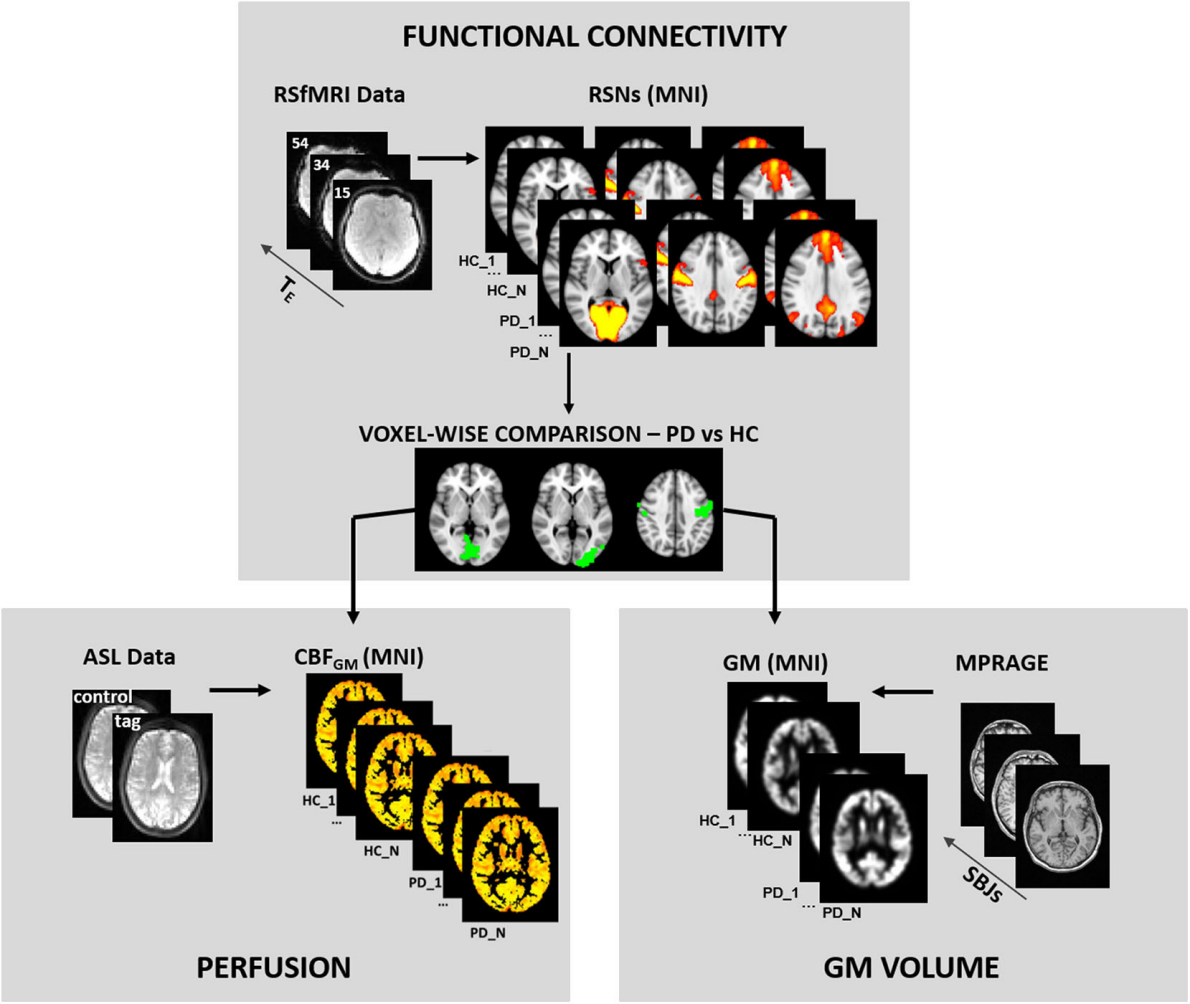
MRI analysis pipeline. The figure shows the pipeline of MRI analyses. Briefly, the preprocessing is shown in each panel for the different modalities. The functional results of the comparison between healthy control (HC) and Parkinson's disease (PD) groups were used as masks of the statistical analysis of perfusion and gray matter maps.

The pipeline of MRI processing is schematized in Figure 1.

#### Pre-processing of MRI Data

##### 3D-T1 MPRAGE

The MPRAGE, which was used as an anatomical reference for registration purposes, was skull-stripped by means of bet toolbox (28), then the SIENAX algorithm (29) was run in order to segment the brain tissues in GM, white matter (WM), and cerebrospinal fluid. A voxel-based morphometry (VBM) analysis (30) was then carried out. Specifically, a symmetrical study-specific template was created in MNI standard space; then, using a non-linear registration, individual GM images were aligned to the study-specific template. Finally, the GM images were spatially smoothed with σ = 3.

##### rsfMRI Dataset

Movement parameters were estimated for each subject-specific ME-rsfMRI dataset by means of FEAT (31). Subjects presenting with relative movement <0.5 mm were excluded from the study. The first 10 volumes (out of 200) were discarded to allow for magnetization stabilization.

The rsfMRI dataset was then preprocessed with the ME-Independent Component Analysis (ICA) algorithm (32, 33). After standard preprocessing comprising motion correction and realignment, the MEICA algorithm performed the estimation of an optimal combination of the three echoes together with a denoising step, based on the TE dependencies, of the ICA-derived components. The denoised volume was then aligned with the subjects' MPRAGE by means of a linear transformation performed using a Boundary-Based registration (BBR) (34, 35). The BBR simultaneously performed the registration and the distortion correction using the acquired field map.

MPRAGE images were normalized to the Montreal Neurological Institute (MNI) atlas using the advanced normalization tools (ANTs; http://stnava.github.io/ANTs) (36) and subsequently used to align the functional data to standard space.

##### ASL Dataset

The preprocessing of the pCASL dataset included realignment and motion correction of the original tag and control volumes, using ANT's software package. The group of 12 tag and control volumes acquired with the same delay was then separately averaged. The estimation and calibration of cerebral blood flow maps were performed by means of oxford_asl and asl_calib tools (37) respectively, by setting the required parameters as follows: T1 of brain tissue = 1.2 s, T1 of blood = 1.36 s, tagging efficiency = 0.8 accordingly (38, 39). Partial volume correction was performed using GM and WM masks derived from the MPRAGE and registered to the ASL space and considering GM perfusion as 2.5 that of WM, as described in Marshall et al. (40). Then, corrected CBF maps were non-linearly registered to the MNI standard space and smoothed with a Gaussian kernel (σ = 3).

#### Group Level Analyses

For group analysis, the rsfMRI datasets were then decomposed in spatial independent components (IC) by means of the MELODIC toolbox (41) setting the dimensionality to 20. The derived IC were visually classified in order to detect the well-defined Smith's resting state networks (RSN) described in Smith et al. (42). Dual regression (43, 44), one on the group spatial maps and one on the subject's time series, was run on the group ICA derived from the functional dataset and allowed to derive subject-specific spatial maps. Then the comparison between the PD and HC groups was implemented through a randomize tool (45) using threshold-free cluster enhancement (TFCE) with 5,000 permutations. Furthermore, in the PD patient group, a partial correlation (age and sex as covariates) between *z*-values extracted from the clusters of significant FC differences and UPDRS III was performed.

In order to understand if the FC changes were accompanied by perfusion or volumetric alterations, we also performed a voxel-wise analysis of CBF and GM volume, comparing PD and HC in the areas of FC differences. The statistics were carried out by means of the randomize tool with 5,000 permutations and cluster detection with TFCE (45), and were restricted within the cluster of significant FC difference between the two groups (Figure 1), using them as masks. The percentage of the altered FC cluster that reported CBF differences was also computed. Finally, in the PD group patients, to test the effect of levodopa treatment on our perfusion results, we assessed the correlation between the CBF values of the significant cluster and LEDD.

## Results

### Sample Demographic and Neuropsychological Evaluation

Demographic data and neuropsychological scores are reported in Table 1 for the two groups. PD patients and HCs were age- and sex-matched at the group level; the clinical phenotype of patients was tremor-dominant in 54% and akinetic-rigid in 46%. Five patients were treated with antidepressant (mirtazapine or escitalopram or duloxetine), and only three patients were taking low dose of benzodiazepine (prazepam or alprazolam). The overall cognitive performance was in the range of normality for both HC and PD. However, the comparison between the MoCA total score of HC and PD revealed a significant reduction in PD (26.4 vs. 24.8, *p* = 0.025) and in the subscores of visuospatial (*p* = 0.002) and memory (*p* < 0.001) functions (Table 1). None of the enrolled subjects was excluded for excessive movements.

**Table 1:** Demographic characteristic of HC and PD groups.

	**HC (*n* = 18)**	**PD (*n* = 26)**	***p*-value**
Males *n* (%)	11 (61%)	22 (85%)	0.077^a^
Age in years, mean (SD)	65.6 (8.25)	66.85 (8.0)	0.62^b^
Disease duration in years, median (IQR)	–	3 (2)	–
UPDRS III, mean (SD)	–	21.92 (13.2)	–
Tremor-dominant/Akinetic-rigid *n* (%)	–	14 (54%)/12 (46%)	–
H&Y, median (IQR)	–	1.5 (1)	–
LEDD, mean (SD)	–	228.2 (139.5)	–
MoCA, median (IQR)	26.43 (3.94)	24.84 (3.73)	**0.025**^**c**^
Visuo-spatial	3.9 (0.72)	3.3 (1.32)	0.002^*^^c^
Executive	3.77 (1)	2.91 (1.46)	0.015^*^^c^
Memory	5 (1)	3 (3)	0.0004^*^^c^
Attention	5.76 (0.89)	5.71 (0.46)	0.892^*^^c^
Language	5.9 (1.34)	5.27 (0.825)	0.037^*^^c^
Orientation	6.04 (0.07)	5.99 (0.31)	0.026^*^^c^

*a = chi-squared test; b = independent sample t-test; c = Mann–Whitney test*.

* *MoCA subscores were corrected for multiple comparison using Bonferroni correction resulting in α = 0.008. MoCA scores were adjusted for age and education when required. SD, standard deviation; IQR, interquartile range; UPDRS III, Unified Parkinson's Disease Rating Scale—Part III; H&Y, Hoehn and Yahr; LEDD, levodopa equivalent daily dose; MoCA, Montreal Cognitive Assessment. Significant p-values are highlighted in bold*.

### Functional Connectivity Results

Eleven ICs out of 20 were classified as RSN according to Beckmann et al. (46) and are reported in Supplementary Figure 1.

**Figure 2:**
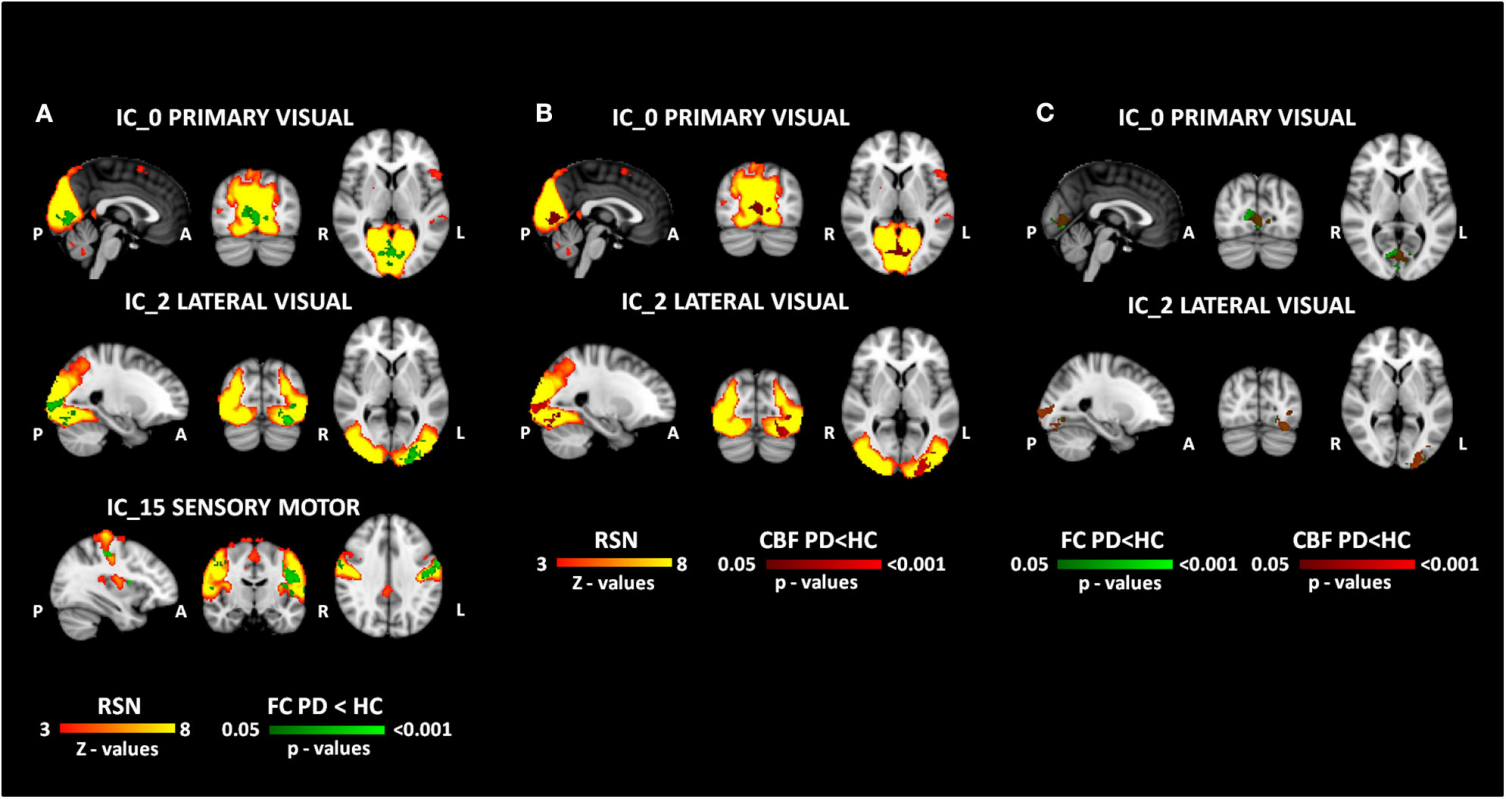
MRI results. The results of functional and perfusion MRI analyses are reported in the figure. **(A)** The functional changes (FC HC > FC PD in green) within the resting state networks (red-yellow). **(B)** The perfusion changes (CBF HC > CBF PD in red) overlapped to the functional network (red-yellow). **(C)** The overlap between functional (green) and perfusion (red) clusters of significant differences. All the reported *p*-values are FWE corrected.

Significantly lower FC was observed for PD patients within the sensory-motor network (p_FWE_ = 0.01) and within the primary (p_FWE_ = 0.022) and lateral (p_FWE_ = 0.01) visual RSNs (Figure 2A). The maximum peak, the extension, and the localization of the clusters of significant FC difference are reported in Table 2.

**Table 2:** Functional connectivity differences.

**RSN**	**Dimension [voxels]**	**Harvard–oxford atlas**	**Position (COG)**			**Minimum *p*-value**	**Peak**		
			**x[mm]**	**y[mm]**	**z[mm]**		**x[mm]**	**y[mm]**	**z[mm]**
Primary visual	605	Lingual G., intracalcarine C.	2.88	−78.1	3.84	0.022	0	−80	−4
Lateral visual	459	Occipital pole	−21.5	−95.7	−1.99	0.018	−20	−102	−8
	302	Occ. fusiform G.	−25.6	−78.8	−14.7	0.01	−30	−78	−18
Sensory motor	968	Post/Pre-central G.	−48	−12.3	36.8	0.01	−64	0	20
	304	Post/Pre-Central G.	61.3	−3.18	22.5	0.02	64	2	20
	103	Post-central G.	50.9	−15.3	49.3	0.03	52	−20	52
	51	Central opercular C., hescl G.	−50.9	−11.8	8.12	0.032	−50	−14	6
	18	Post/Pre-Central G.	−26.7	−27.2	60.3	0.024	−26	−26	60
	13	Inferior frontal G.	−40	25.2	22.1	0.028	−40	24	20
	11	Post/Pre-central G.	37.6	−20	57.5	0.04	98	−20	58
	10	Precentral G, middle frontal G.	−39.4	−3.2	57.2	0.02	−38	−4	56

*Cluster of significant differences (HC > PD) in functional connectivity within resting state networks. Only the cluster with an extension ≥10 voxels are shown in the table. The coordinates reported refer to MNI standard space. RSN, resting state network; COG, center of gravity; G, gyrus; C, cortex*.

Furthermore, the partial correlation showed a negative trend (*p* = 0.06, r = −0.38) between the *z*-values of the sensory-motor network and the UPDRS III score for PD patients.

### Perfusion Results

Perfusion alterations were found both within primary (p_FWE_ = 0.037) and lateral (p_FWE_ = 0.014) visual networks, whereas no difference in CBF was detected in the sensory-motor one (Figure 2B). The maximum peak, the extension, and the localization of the clusters of CBF significant difference are reported in Table 3. The percentage of the overlap resulted in 46% and 74% of voxels for primary and lateral visual components, respectively (Figure 2C). The alteration in CBF did not correlate with any clinical variable, and no significant relationship between the CBF values of the significant CBF clusters and LEDD variables was found (*r* = 0.063, *p* = 0.763 for primary visual network and *r* = 0.141, *p* = 0.502 for the lateral visual one).

**Table 3:** Perfusion differences.

**RSN**	**Dimension [voxels]**	**Harvard–oxford atlas**	**Position (COG)**			**Minimum *p*-value**	**Peak**		
			**x[mm]**	**y[mm]**	**z[mm]**		**x[mm]**	**y[mm]**	**z[mm]**
Primary visual	279	Lingual G., intracalcarine C.	−1.15	−77.5	3.23	0.037	−6	−88	−4
Lateral visual	328	Occipital pole	−24.5	−95.4	−0.58	0.014	−22	−104	0
	247	Occipital fusiform G.	−27.4	−78.6	−15.6	0.019	−34	−78	−16

*Cluster of significant differences (HC > PD) of perfusion within the cluster of functional connectivity changes. Only the cluster with an extension ≥10 voxels are shown in the table. The coordinates reported refer to MNI standard space. RSN, resting state network; COG, center of gravity; G, gyrus; C, cortex*.

### VBM Results

No GM atrophy was found concurrent with the functional alteration for our cohort of early PD patients when compared to HC.

## Discussion

The present work aimed to study the relationship between changes in FC and altered perfusion reported in PD. To do so, we concurrently assessed FC and CBF in a cohort of early PD patients and HC by means of rsfMRI and ASL, respectively. FC changes were found in the sensory-motor and visual cortices of our PD patients. Interestingly, the FC alterations within the visual cortex were also accompanied by altered CBF.

The significant FC reduction that we observed in the SMN extended between pre- and post-central gyri and also comprised part of the middle frontal gyrus. Consistently, other studies using an ICA-based approach had previously evidenced changes in FC specific to the SMN in resting conditions. Canu et al. showed a decreased connectivity within the SMA and primary motor cortex, belonging to the SMN, in PD compared to HC (47), confirming previous findings of reduced SMA FC (48). Moreover, the aberrant FC pattern that we detected in our sample was linked to symptom severity measured using MDS-UPDRS III. We hypothesized that the altered SMN FC might be related to a disconnection of the striatal pathways following dopaminergic denervation. In fact, paralleling the neuropathological progression of PD, decreased FC between cortical and subcortical motor areas involving the dopaminergic corticostriatal loop has been reported (49).

In this study a significant FC decrease was also observed in primary (lingual gyrus and intracalcarine cortex) and lateral (inferior lateral occipital cortex, specifically occipital pole, and fusiform gyrus) VNs. The key regions of the primary visual areas are related to visual awareness, whereas the secondary visual network is involved in visual experience (50). Our findings point to dysfunction not only of the primary visual system but also of higher visual processing areas in the extrastriate cortex. The decreased activity of the primary visual network is probably due to specific PD-associated retinopathy targeting the striate visual areas (51). The functional alteration of the extrastriate visual pathways is supported by the significantly lower visuospatial performances (as assessed using MoCA subscales) that were found in our PD patients with respect to HC.

Similar FC changes have already been reported in literature (11, 52). Interestingly, the decreased connectivity within the primary and lateral VNs was accompanied by a significant CBF reduction. Our perfusion results are in line with previous studies quantitatively investigating vascular alteration in PD by means of ASL (16, 17, 53) or other modalities (14). Specifically, Melzer and colleagues reported preserved perfusion in post- and pre-central gyri, while perfusion was reduced in the posterior parieto-occipital cortex, similarly to Syrimi et al. (53). Abe et al. (14) also found reduced CBF in the same region by means of single-photon emission computed tomography.

The functional and perfusion changes were extensively concurrent in the visual RSN, with an overlap ranging from 46% to 74% in our PD sample. The hypoperfusion that we found in occipital areas concurrently to a decrease in FC may be indicative of a possible impairment of the NVC mechanism in PD. It has been previously proposed that CBF reductions might be due to modifications of non-dopaminergic transmitter systems (specifically cholinergic, serotoninergic, and noradrenergic) and their neurovascular innervation of the neocortex (16, 18, 19, 54). Contextually, Shimada et al. (55) reported an alteration of the cholinergic system in PD patients without dementia, which was most significant in the medial occipital cortex. According to Braak staging (56), neuronal cholinergic degeneration occurs at the same stage of nigral pathology, which characterizes relatively early phases of the disease. Thus, we hypothesize that structural and microstructural changes in the noradrenergic and cholinergic system nuclei at this stage of the pathology may be the cause of the alteration of the coupling between neural activity and blood flow observed in visual areas of our early PD patients [H&Y median (IQR), 1.5 (1)].

Furthermore, the FC and CBF alterations in VNs were not accompanied by local GM atrophy, suggesting that functional changes occur prior to tissue loss. Evidence from recent studies reporting CBF reductions at the early stage of the disease in cortical regions without manifested pathology (16, 57) supports this hypothesis. Moreover, Fernandez-Seara et al. (16) demonstrated that there is not a direct correspondence between GM atrophy and hypoperfusion, with the latter being more extensive throughout the brain.

One of the main drawbacks regarding FC studies in PD is the heterogeneity of the results presented in the literature, likely due to clinically variegated samples of patients and different methodological approaches. For these reasons, in the present study, we used a well-established data-driven ICA-approach together with a dual regression analysis. This method investigates all the GM voxels and exploits the simultaneous analysis of several subjects, thus increasing the signal-to-noise ratio (58). This study is not without limitations. First, a 1.5T field scanner was employed for data acquisition. Despite being extensively used in the clinical setting, 1.5T MRI has relatively low signal-to-noise ratio. For this reason, both the rsfMRI and ASL sequences were set to partially mitigate this problem. Specifically, a multi-echo rsfMRI sequence was employed to improve the image contrast and to reduce image distortions by means of an optimal combination of the volumes acquired at three different echo times (32). For what concerns the ASL sequence, a pseudocontinuous acquisition scheme was used, together with a background suppressed 3D gradient and spin echo readout, aiming to enhance both SNR and signal tagging efficiency, as recommended by international guidelines (59). Another limitation of the study is the relatively small size of the sample, which may have prevented us from showing significant correlations between clinical and neuroimaging parameters. Furthermore, levodopa and benzodiazepines/antidepressants may interfere with fMRI (60, 61) and CBF analysis (62, 63). However, a group of clinically homogeneous patients, under stable pharmacological treatment, was considered, and when we tested the effect of levodopa treatment on our perfusion results, no significant relationship between the variables was found. Finally, only MoCA scores and subscales were available for our sample, so the addition of more precise neuropsychological tests should be considered.

Altogether, our results suggest that MRI-derived measures, such as FC and CBF, may constitute valuable biomarkers to detect early neurovascular dysfunction occurring in PD prior to structural modification in terms of GM atrophy. Since FC and CBF provide complementary information about the neurovascular unit physiology, concurrently assessing both of them is crucial. Multimodal longitudinal studies are warranted to better understand the evolution of neurovascular dysfunction along with PD disease progression and to monitor treatment-related changes due to pharmacological and/or rehabilitative interventions.

## Data Availability Statement

The datasets generated for this study are available on request to the corresponding author.

## Ethics Statement

The studies involving human participants were reviewed and approved by Fondazione Don Carlo Gnocchi Local Ethics Committee. The patients/participants provided their written informed consent to participate in this study.

## Author Contributions

ML, AP, and FB contributed to the conception and design of the study. AP, NB, and LP performed the MRI data analysis. AP and ML performed the statistical analyses. AP wrote the first draft of the manuscript. FR and SD performed the neuropsychological evaluation. RN, FB, and MM recruited subjects and performed the clinical evaluation. All authors participated in reviewing the work, providing important intellectual content, and approving the final form.

## Conflict of Interest

The authors declare that the research was conducted in the absence of any commercial or financial relationships that could be construed as a potential conflict of interest.
