# Supplementary figures and images for: Multimodal Evaluation of Neurovascular Functionality in Early Parkinson's Disease
Source: Front Neurol. 2020 Aug 26;11:831. doi: 10.3389/fneur.2020.00831 (PMC7479303; doi:10.3389/fneur.2020.00831)

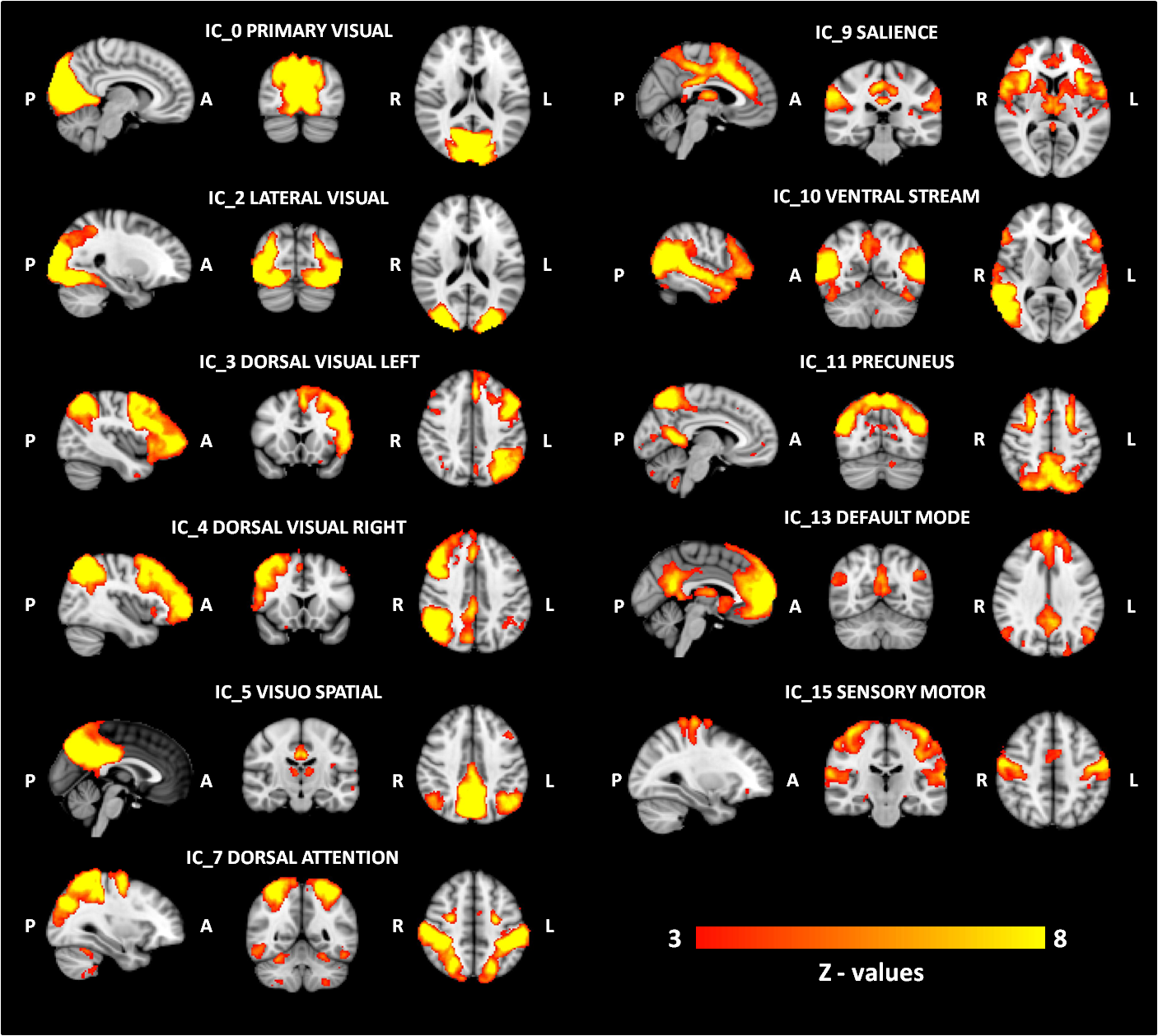

Supplement: Supplementary file 1 [file Image_1.TIFF]
