# Supplementary material for: Multimodal Evaluation of Neurovascular Functionality in Early Parkinson's Disease
Source: Front Neurol. 2020 Aug 26;11:831. doi: 10.3389/fneur.2020.00831 (PMC7479303; doi:10.3389/fneur.2020.00831)
Supplement: Supplementary file 2 [file Table_1.DOCX]

**Supplementary Figure 1 - Resting State Networks.** The figure represents the estimated independents components that have been classified as RSN (11 out of 20) according to Smith's template.
